# Metabolic pathway analysis of hyperuricaemia patients with hyperlipidaemia based on high-throughput mass spectrometry: a case‒control study

**DOI:** 10.1186/s12944-022-01765-0

**Published:** 2022-12-31

**Authors:** Xue Wei, Xiaodong Jia, Rui Liu, Sha Zhang, Shixuan Liu, Jing An, Lei Zhou, Yushi Zhang, Yuanning Mo, Xiao Li

**Affiliations:** 1 Tianjin Union Medical Center, Tianjin Medical University, Tianjin, 300070 China; 2 Tianjin Union Medical Centre, Tianjin, 300121 China; 3 Tianjin Yunjian Medical Technology Co., Ltd., Tianjin, China

**Keywords:** Hyperuricaemia, Hyperlipidaemia, Metabolomics, Amino acid metabolism, Fatty liver, Chronic kidney disease

## Abstract

**Background:**

Both hyperuricaemia and hyperlipidaemia are common metabolic diseases that are closely related to each other, and both are independent risk factors for the development of a variety of diseases. HUA combined with hyperlipidaemia increases the risk of nonalcoholic fatty liver disease and coronary heart disease. This study aimed to investigate the relationship between HUA and hyperlipidaemia and study the metabolic pathway changes in patients with HUA associated with hyperlipidaemia using metabolomics.

**Methods:**

This was a case‒control study. The prevalence of hyperlipidaemia in HUA patients in the physical examination population of Tianjin Union Medical Centre in 2018 was investigated. Metabolomics analysis was performed on 308 HUA patients and 100 normal controls using Orbitrap mass spectrometry. A further metabolomics study of 30 asymptomatic HUA patients, 30 HUA patients with hyperlipidaemia, and 30 age-and sex-matched healthy controls was conducted. Differential metabolites were obtained from the three groups by orthogonal partial least-squares discrimination analysis, and relevant metabolic pathways changes were analysed using MetaboAnalyst 5.0 software.

**Results:**

The prevalence of hyperlipidaemia in HUA patients was 69.3%. Metabolomic analysis found that compared with the control group, 33 differential metabolites, including arachidonic acid, alanine, aspartate, phenylalanine and tyrosine, were identified in asymptomatic HUA patients. Pathway analysis showed that these changes were mainly related to 3 metabolic pathways, including the alanine, aspartate and glutamate metabolism pathway. Thirty-eight differential metabolites, including linoleic acid, serine, glutamate, and tyrosine, were identified in HUA patients with hyperlipidaemia. Pathway analysis showed that they were mainly related to 7 metabolic pathways, including the linoleic acid metabolism pathway, phenylalanine, tyrosine and tryptophan biosynthesis pathway, and glycine, serine and threonine metabolism pathway.

**Conclusions:**

Compared to the general population, the HUA population had a higher incidence of hyperlipidaemia. HUA can cause hyperlipidaemia. by affecting the metabolic pathways of linoleic acid metabolism and alanine, aspartate and glutamate metabolism. Fatty liver is closely associated with changes in the biosynthesis pathway of pahenylalanine, tyrosine, and tryptophan in HUA patients with hyperlipidaemia. Changes in the glycine, serine and threonine metabolism pathway in HUA patients with hyperlipidaemia may lead to chronic kidney disease.

## Background

Uric acid is a metabolite of purine. Disorder of purine metabolism,makes uric acid synthesis increase and/or excretion decrease, causes hyperuricaemia (HUA) [1]. With the improvement of living standards and changes in dietary structure, the incidence of HUA is increasing each year, and the age of onset is trending toward a younger age [2, 3]. HUA has become the "fourth most common" disease after hypertension, hyperglycemia, and hyperlipidaemia [4]. Most recent studies have shown a strong association between HUA and hypertension, nonalcoholic fatty liver disease (NAFLD), chronic kidney disease (CKD), cardiovascular disease, cerebrovascular disease, metabolic syndrome and other diseases [5–7]. Hyperlipidaemia, a common metabolic disease, is also closely connected with NAFLD, CKD and metabolic syndrome and other diseases [8–10]. An epidemiological survey in Tianjin, China, in 2015 showed that 60% of HUA patients had hyperlipidaemia [4]. An epidemiological survey in Guangdong, China, in 2016 showed that the combined hyperlipidaemia rate of patients with hyperuricaemia was 64.1%, which was significantly higher than that of patients without hyperuricaemia (20.0%) [11]. Guo J et al.'s epidemiological survey of 6,382 people examined in Qinghai Province, China, identified HUA as a potential risk factor for hyperlipoproteinaemia and hypertriglyceridaemia [12]. In addition, HUA combined with hyperlipidaemia will further lead to metabolic syndrome, NAFLD, coronary heart disease, and other diseases [13–15], causing a burden to patients and families. Currently, reports in the literature on the relationship between HUA and hyperlipidaemia mostly perform correlation analysis via case–control studies. There are few reports on the pathogenesis of HUA with hyperlipidaemia.

Metabolomics is an omics technology that has emerged in recent years; it uses mass spectrometry to detect small molecules in biological samples [16]. It has played a vital role in studying the changes of endogenous small molecules and revealing individual metabolic characteristics [17]. It has been widely used in the exploration of disease mechanisms, the screening of diagnostic markers, and the monitoring of drug efficacy [18–20]. This technology also provides a new platform for exploring the relationship between HUA and hyperlipidaemia and has broad application prospects and practical significance.

The purpose of this study was to investigate the relationship between HUA and hyperlipidaemia from a metabolic pathway perspective and to study the metabolic pathways changes in patients with HUA with hyperlipidaemia.

## Materials and methods

### Study design

In this study, serum metabolites of HUA and HUA patients with hyperlipidaemia were analysed by ultrahigh-performance liquid chromatography-tandem mass spectrometry (UHPLC‒MS) using case–control study design, and changes in metabolic pathways were investigated based on differential metabolites.

### Study participants

In this study, the prevalence of HUA and hyperlipidaemia in HUA patients was investigated in population who received a physical examination at Tianjin Union Medical Centre in 2018. Serum metabolite analysis using Orbitrap mass spectrometry was performed on 308 HUA patients and 100 normal controls examined between 25 April and 19 September 2018. Participants were selected to exclude the influence of disease and other factors on the metabolomics analysis. According to the inclusion criteria, the experimental group of this study was comprised of patients with the following characteristics: male serum uric acid (SUA) > 420 mol/L, female SUA > 360 mol/L, fasting plasma glucose (FPG) < 7.0 mmol/L, estimated glomerular filtration rate (eGFR) > 90 mL/(min·1.73 m2), alanine aminotransferase (ALT) < 40 U/L, aspartate aminotransferase (AST) < 40 U/L, and no history of hypertension, coronary heart disease and other cardiovascular and cerebrovascular diseases, tumours, and major diseases. Patients with triglycerides (TG) < 5.2 mmol/L and total cholesterol (TC) < 1.7 mmol/L constituted the asymptomatic HUA (HUA) group. Patients with TG ≥ 5.2 mmol/L and/or TC ≥ 1.7 mmol/L constituted the HUA with hyperlipidaemia (HUA + HPA) group. According to age and sex, 30 healthy people who received physical examinations in Tianjin Union Medical Centre in 2018 were matched as the experimental control group. Finally, a total of 90 participants formed the cohort of this study, including 30 normal control (NC) individuals (24 males and 6 females, average age 36.23), 30 patients in the HUA group (24 males and 6 females, average age 34.77), and 30 patients in the HUA + HPA group (25 males and 5 females, average age 36.03).

### Blood sampling

Fasting blood samples were collected from all participants in this study according to conventional methods. These samples were immediately centrifuged to separate the serum. The levels of serum creatinine (Scr), blood urea (BUN), FPG, TC, TG, low-density lipoprotein cholesterol (LDLc), high-density lipoprotein cholesterol (HDLc), very low-density lipoprotein cholesterol (VLDLc), SUA and other biochemical indicators were measured using an ARCHITECT 16,000 automatic biochemical analyser (Abbott, USA). The levels of serum TC, TG, FPG, SUA were determined using Roche's reagents and calibrators, the levels of serum LDL-C, HDL-C were determined using Wittman Biotech's reagents and the Roche calibrators. All samples were collected from the top serum after completion of biochemical testing on the same day as blood collection and stored in the refrigerator at -80 • C for uniform mass spectrometry analysis.

### Metabolomics analysis

#### Experimental equipment and quality control

Thermo Vanquish UHPLC was selected for the liquid phase system, and Exactive Plus Orbitrap (both San Jose, CA, USA) was selected for the mass spectrometer to accomplish the metabolomics experiment. The mass spectrometer was set to positive ionization mode, and detailed parameters and settings can be found in the article by Du B et al. [20]. Sigma quality control serum from human serum matrix was selected as the quality control product for this study with coefficient of variation ≤ 15%, and each of the nine samples was matched with quality control serum.

#### Experimental procedure

Validation of quality control was performed by calculating a coefficient of variation ≤ 15% of total ion flow chromatography values for all QC sera inserted across the entire run following online assays. Ten microliters of thawed serum from the − 80 °C freezer and 240 μL of extraction buffer (methanol/acetonitrile/water, 5:3:2, v/v/v) that was prechilled at -20 °C were added into 1.5 ml Eppendorf (EP) tubes to extract serum metabolites. Mixtures were mixed in a continuous vortex for 30 min at 4 °C, and their supernatants were transferred to new EP tubes. Following the transfer of the supernatants, they were centrifuged at 12,000 g at 4 °C for 30 min. The final step was to transfer the supernatant from each sample (170 μL) to autosampler vials and prefreeze them at -20 °C for at least two hours prior to UHPLC‒MS analysis.

### General data processing

SPSS.24.0 was used to analyse the general physical information and biochemical indicators of the subjects, and *P* < 0.05 indicated statistical significance.

### Mass spectrometry data processing

#### Data processing

Referring to the Du B et al. processing flow, mass spectral data were extracted [20]. Peak alignment, peak correction, normalization and other operations were performed on mass spectral peaks.

#### Data annotation

Annotation of the mass spectral data was based on the Human Metabolome Database (HMDB, https://hmdb.ca/) and Kyoto Encyclopedia of Genes and Genomes (KEGG, https://www.genome.jp/kegg/) databases using the xMSannotator software package.

#### Univariate analysis

Fold changes and *P* values were calculated for normalized peak groups.

#### Multivariate analysis

R software was used to construct principal component analysis (PCA) and orthogonal partial least-squares discrimination analysis (OPLS-DA) models. Metabolites with variable importance in the projection (VIP) > 1 and *P* < 0.05 in the OPLS-DA model were selected as differential metabolites.

#### Metabolic pathway analysis

MetaboAnalyst 5.0 (http://www.metaboanalyst.ca) software was used to analyse the metabolic pathways in accordance with the differential metabolites, and the pathways were considered significant when Impact > 0.1 and *P* < 0.05.

## Results

### Epidemiological results

A total of 17,307 cases (10,539 males and 6,768females) were investigated in 2018, including 3,311 HUA patients (2,748males and 563females, average age 41.75). HUA prevalence was 19.1% (26.1% for males and 8.3% for females). Biochemical lipids were tested in 3,108 HUA patients, 2,155 of whom had hyperlipidaemia, the prevalence of hyperlipidaemia in HUA patients was 69.3%.

### Physical examination and serum biochemical results of the metabolomic participants

The physical examination and serum biochemical results of the participants are shown in Table 1. The results showed that there were significant differences in body mass index (BMI) among the three groups. The diastolic blood pressure of the HUA + HPA group was significantly different from that of the other two groups. However, the BMI and diastolic blood pressure of most participants were at normal levels or were slightly increased. There were no statistically significant differences in age, sex, HDLc, BUN, Scr, FPG, or eGFR among the three groups. The serum levels of SUA were higher in the two groups than in the NC group, and there was no statistically significant between the two groups of patients. The serum levels of TC, TG, LDLc, VLDLc, ALT and AST in the HUA + HPA group were significantly higher than those in the other two groups, and there was no significant difference between the HUA group and the NC group. However, the ALT and AST levels in the HUA + HPA group were still within the reference value range. The medical history investigation found that most of the patients in the HUA + HPA group had a history of fatty liver.

**Table 1:** Physical examination and serum biochemical results of the metabolomic participants

Parameters	NC (*n* = 30)		HUA (*n* = 30)		HUA + HPA (*n* = 30)	
	‾X	SD	‾X	SD	‾X	SD
Sex (M/F)	24/6	/	24/6	/	25/5	/
Age (years)	36.23	10.01	34.77	6.23	36.03	5.80
BMI (kg/m^2^)	22.74	3.61	24.92	3.36*	27.83	3.14^#&^
SBP (mmHg)	120.67	14.52	120.73	15.61	126.80	12.51
DBP (mmHg)	76.33	8.15	78.87	9.79	87.90	9.78^#&^
SUA (μmol/L)	324.77	69.55	466.10	69.44*	472.83	59.43^#^
FPG (mmol/L)	5.00	0.40	5.22	1.27	5.16	0.50
ALT (U/L)	12.97	7.13	19.10	9.72	36.60	24.39^#&^
AST (U/L)	16.70	4.33	18.10	4.33	24.16	11.39^#^
TC (mmol/L)	4.12	0.39	4.44	0.53	5.77	0.69^#&^
TG (mmol/L)	0.91	0.31	1.08	0.29	3.05	1.70^#&^
HDLc (mmol/L)	1.28	0.19	1.32	0.31	1.30	0.28
LDLc (mmol/L)	2.67	0.38	2.69	0.76	3.55	0.80^#&^
VLDLc (mmol/L)	0.41	0.14	0.49	0.13	1.39	0.77^#&^
BUN (μmol/L)	4.48	0.93	4.81	1.13	4.95	1.11
Scr (mmol/L)	70.53	10.33	75.40	12.18	74.00	11.78
eGFR mL/(min·1.73 m^2^)	112.76	10.58	109.12	10.92	109.64	9.87

*SBP* Systolic blood pressure, *DBP* Diastolic blood pressure, *BMI* Body mass index

^*^ *P* < 0.05 for NC vs. HUA

^#^ *P* < 0.05 for NC vs. HUA + HPA

^&^ *P* < 0.05 for HUA vs. HUA + HPA

### Serum metabolite profiles of participants in the NC, HUA and HUA + HPA groups

**Fig. 1:**
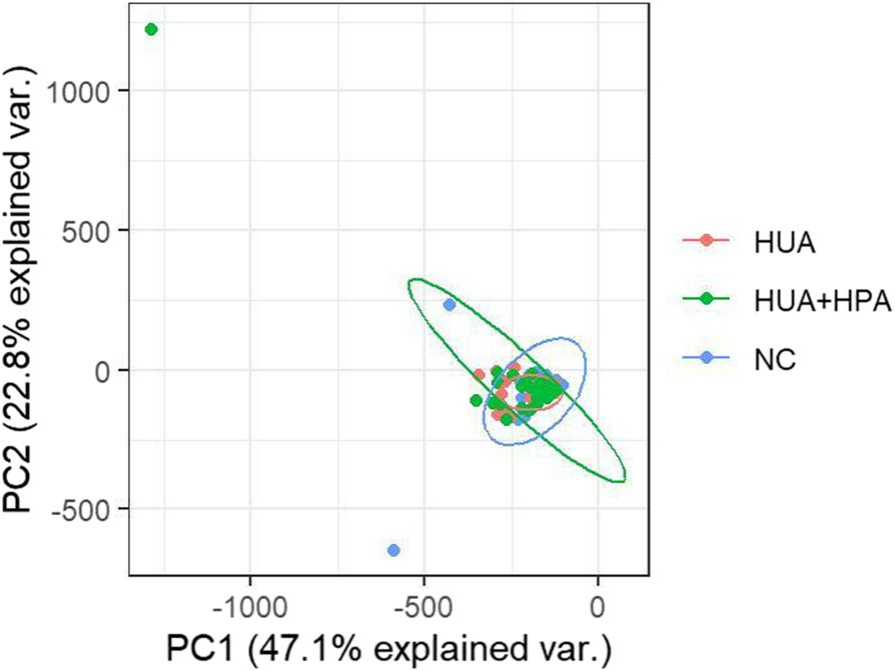
PCA score chart of the three groups

**Fig. 2:**
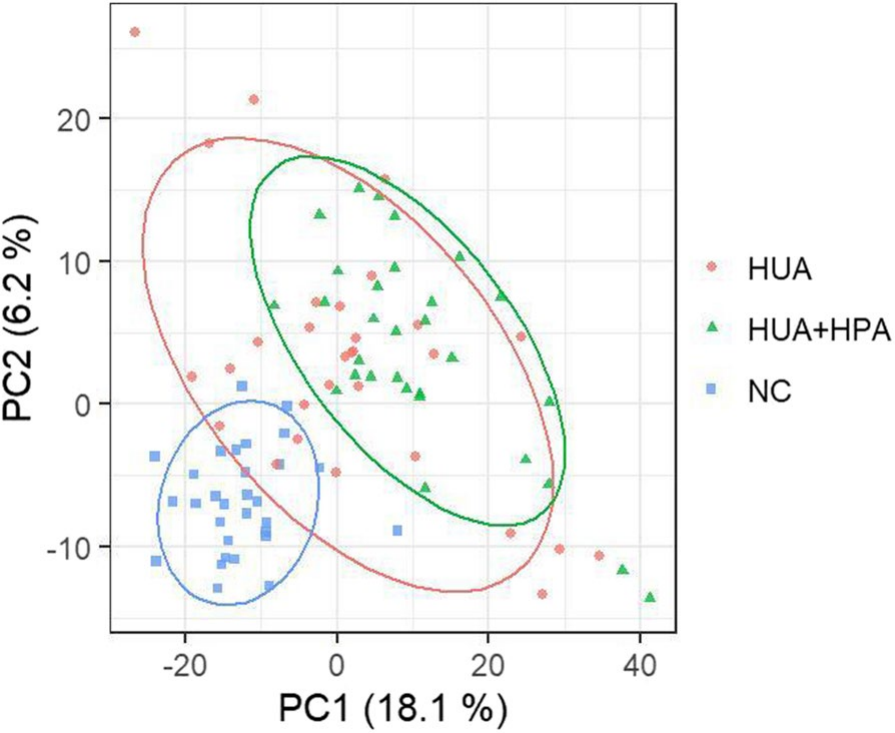
PLS-DA score chart of the three groups

UHPLC‒MS analysis detected a total of 2128 mass spectral peaks in the serum samples of the NC group and the experimental group. After normalizing the data and filling in missing values, 1557 characteristic mass spectral peaks were obtained. A total of 633 metabolites were obtained after annotating the mass spectrum peaks according to the HMDB and KEGG databases. A preliminary analysis of the metabolic characteristics of the subjects was carried out using PCA, and the score chart is shown in Fig. 1. Then, partial least-squares discrimination analysis (PLS-DA) and OPLS-DA were performed, and the PLS-DA score chart is shown in Fig. 2. As shown in the figure, the classification effect of PLS-DA is better than that of PCA.

### Metabolic profile analysis of the NC group and HUA group

**Fig. 3:**
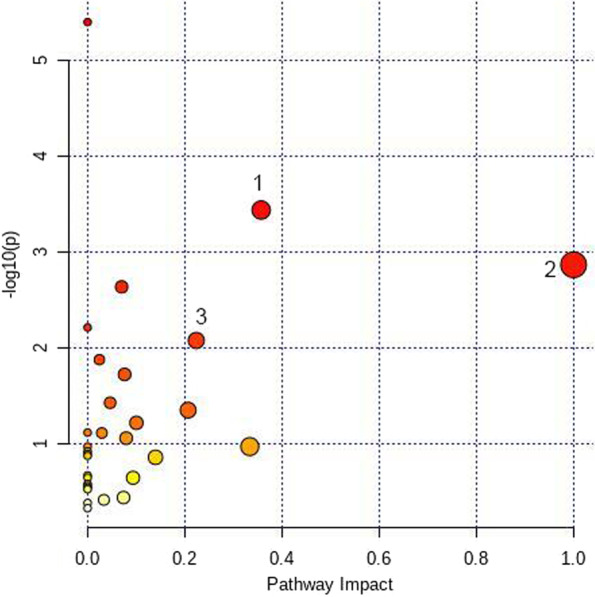
Metabolic pathway map of the NC group and HUA group

According to the OPLS-DA results, 33 differential metabolites were selected. Among them, 26 metabolites, including uric acid, creatinine, and alanine, were upregulated, and 7 metabolites, including arachidonic acid, were downregulated in the HUA group compared to the NC group. Metabolic pathway analysis showed that the differential metabolites were mainly related to three pathways. The detailed results are shown in Fig. 3.

### Metabolic profile analysis of the NC group and HUA + HPA group

**Fig. 4:**
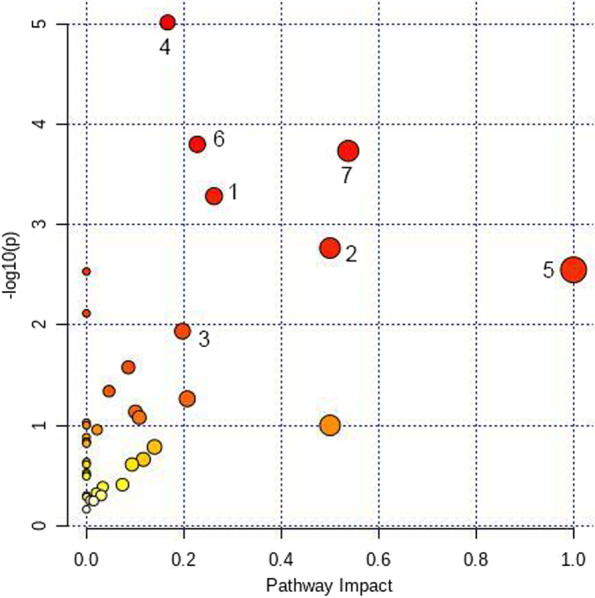
Metabolic pathway map of the NC group and HUA + HPA group

According to the OPLS-DA results, 38 differential metabolites were selected. Among them, 28 metabolites, including uric acid, glutamate, and leucine, were upregulated, and 10 metabolites, including serine and glycine, were downregulated in the HUA + HPA group compared to the NC group. Metabolic pathway analysis showed that the differential metabolites were mainly related to seven pathways. The detailed results are shown in Fig. 4.

### Metabolic profile analysis of the HUA group and HUA + HPA group

**Fig. 5:**
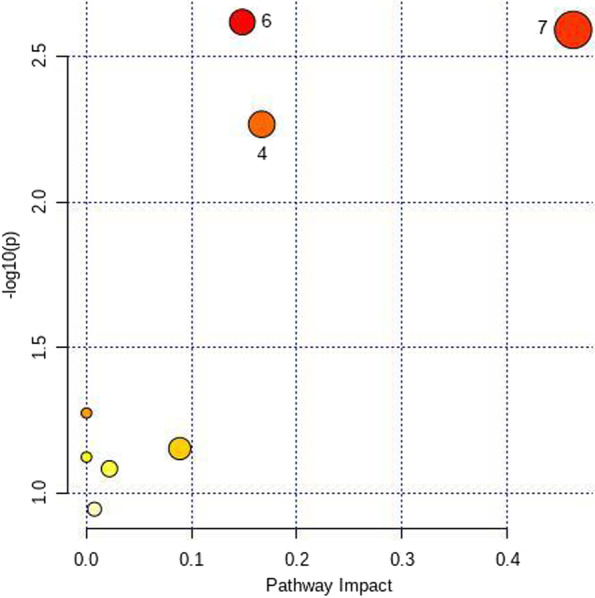
Metabolic pathway map of the HUA group and HUA + HPA group. Note: 1 phenylalanine metabolism, 2 phenylalanine, tyrosine and tryptophan biosynthesis, 3 alanine, aspartate and glutamate metabolism, 4 aminoacyl-tRNA biosynthesis, 5 linoleic acid metabolism, 6 glyoxylate and dicarboxylate metabolism, 7 glycine, serine and threonine metabolism

According to the OPLS-DA results, 4 differential metabolites were selected. Among them, arachidonic acid glycerol and prostaglandin were upregulated, and glycine and serine were downregulated in the HUA + HPA group compared to the HUA group. Metabolic pathway analysis showed that the differential metabolites were mainly related to three pathways. The detailed results are shown in Fig. 5.

## Discussion

### Relationship between HUA and hyperlipidaemia

A preliminary investigation found that 69.3% of the 3108 HUA patients enrolled in this study had hyperlipidaemia, which is higher than the incidence of hyperlipidaemia in the general population (42.6%) [21], and consistent with several large epidemiological findings in China [11, 12]. This finding suggests that HUA could increase the risk of hyperlipidaemia. It is generally accepted in academia that elevated uric acid levels induce reactive oxygen species (ROS) production, leading to oxidative stress [22]. The study by Johnson RJ et al. [23] suggests that oxidative stress can affect the activity of certain enzymes in the Krebs cycle and β-fatty acid oxidation, disrupt fatty acid oxidation, and cause abnormal lipid metabolism. This statement is only one of the inferences that uric acid affects lipid metabolism, and this study complements this statement by using mass spectrometry to analyse linoleic acid metabolism and amino acid metabolism changes in patients with HUA and HUA combined with hyperlipidaemia.

### HUA causes hyperlipidaemia by affecting the linoleic acid metabolic pathway

**Fig. 6:**
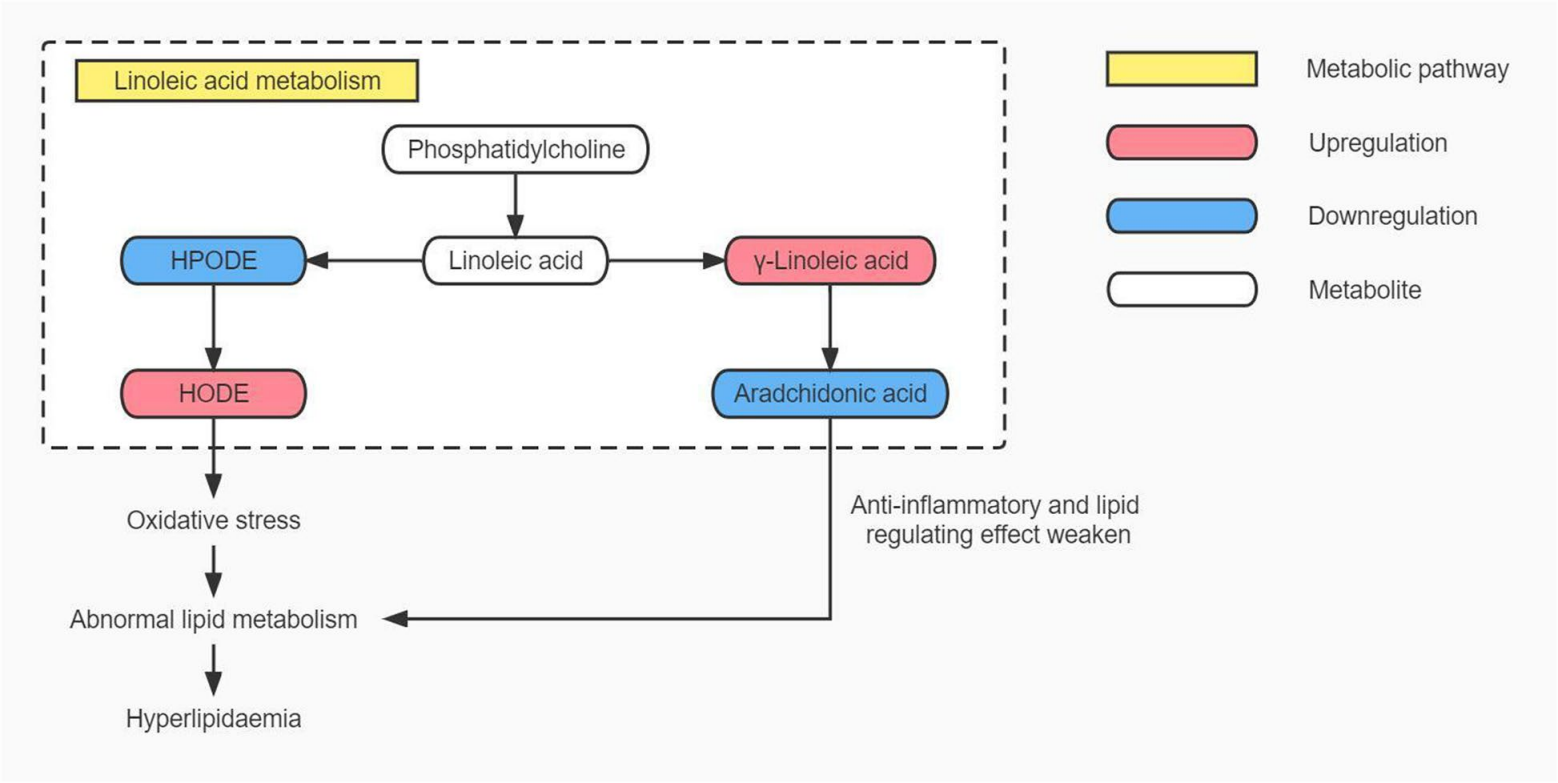
Map of linoleic acid metabolism pathway in HUA patients. Note: HPODE: hydroperoxyoctadecadienoic acid; HODE: hydroxyoctadecadienoic acid. Both HOPDE and HODE are linoleic acid metabolites, and HPODE is unstable and rapidly transforms into HODE upon production.

This study showed significant changes in serum levels of various linoleic acid metabolites in HUA patients when serum TG and TC levels were normal. Similar changes were observed in patients with HUA associated with hyperlipidaemia, further contributing to abnormalities in the linoleic acid metabolism pathway, which were also identified in a metabolomics study of HUA patients by Qin N et al. [24]. In the linoleic acid metabolism pathway, linoleic acid is converted to hydroxyoctadecadienoic acid (HODE) via the lipoxygenase pathway which is associated with oxidative stress [25]. Linoleic acid can also be converted to gamma linoleic acid by the use of 6-acyl coenzyme A dehydrogenase, which further converts to arachidonic acid (AA) for further metabolism [26]. Linoleic acid metabolism and its related arachidonic acid metabolism, as part of fatty acid oxidation, are associated with abnormal lipid metabolism in blood [27]. In this study, HUA induced oxidative stress, interfered with fatty acid oxidation and altered linoleic acid metabolic pathway. On the one hand, this pathway alterations lead to elevated HODE level in HUA patients, exacerbates oxidative stress, further interferes with fatty acid oxidation, and results in abnormal lipid metabolism in blood. On the other hand, changes in this pathway also result in decreased AA level, which weakens the whole-body regulation of blood lipids and anti-inflammatory effects [24, 28], leading to abnormal lipid metabolism, resulting in fat accumulation and hyperlipidaemia. See Fig. 6 for details.

### HUA causes hyperlipidaemia by affecting the alanine, aspartate and glutamate metabolism pathway

**Fig. 7:**
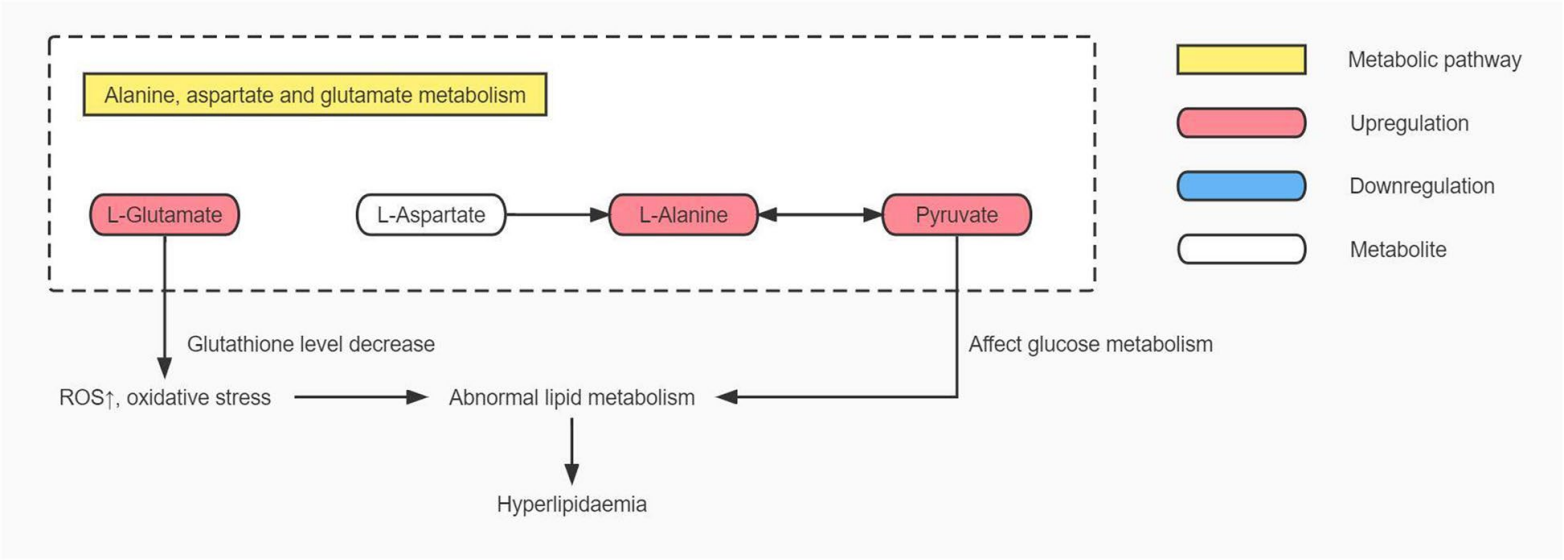
Map of alanine, aspartate and glutamate metabolism pathway in HUA patients

In this study, serum alanine and pyruvate levels were significantly increased in HUA and HUA patients with hyperlipidaemia, and serum glutamate level was significantly increased in HUA patients with hyperlipidaemia. Luo Y et al. [29] and Mahbub MH et al. [30] also found elevated plasma alanine levels in asymptomatic HUA patients. As a result of these changes in the metabolism pathway of alanine, aspartate and glutamate. This pathway is not only associated with important physiological processes such as glycolysis, glycoheterogenesis and tricarboxylic acid cycling [29],but also inhibits the glutamate-cysteine reverse transport system through glutamate, leading to a decrease in glutathione levels in cells, causing elevated reactive oxygen species and exacerbating oxidative stress [31]. In HUA patients changes in the alanine, aspartate and glutamate metabolism pathway may affect physiological processes, such as glucose metabolism and glutathione metabolism, aggravate oxidative stress, disturb fatty acid oxidation, and cause hyperlipidaemia. See Fig. 7 for details.

### Metabolic pathways changes in HUA patients with hyperlipidaemia

As common metabolic diseases, HUA and hyperlipidaemia are closely connected with the occurrence of cardiovascular and cerebrovascular diseases and metabolic syndrome [5, 7, 10]. HUA with is common in clinic [4], but there is no clear conclusion on how the combination of the two causes the occurrence of subsequent diseases. Metabolomics was used to analyse metabolic pathways alterations in HUA patients with hyperlipidaemia, and found that amino acid metabolism pathways alterations were strongly associated with subsequent disease development.

### The relationship between changes in the phenylalanine, tyrosine and tryptophan biosynthesis pathway and fatty liver in HUA patients with hyperlipidaemia

This study showed a significant increase in serum levels of tyrosine and phenylalanine in HUA patients and in serum level of phenylalanine in HUA patients with hyperlipidaemia, resulting in changes in the biosynthesis pathway of phenylalanine, tyrosine and tryptophan. Changes in this pathway were also observed in HUA rat models constructed by Huang et al. [32] and Qin et al. [24] in patients with HUA. The biosynthesis pathway of phenylalanine, tyrosine, and tryptophan is not only strongly associated with the development of HUA but also with nonalcoholic fatty liver disease [33]. HUA and hyperlipidaemia are independent risk factors for NAFLD.

**Fig. 8:**
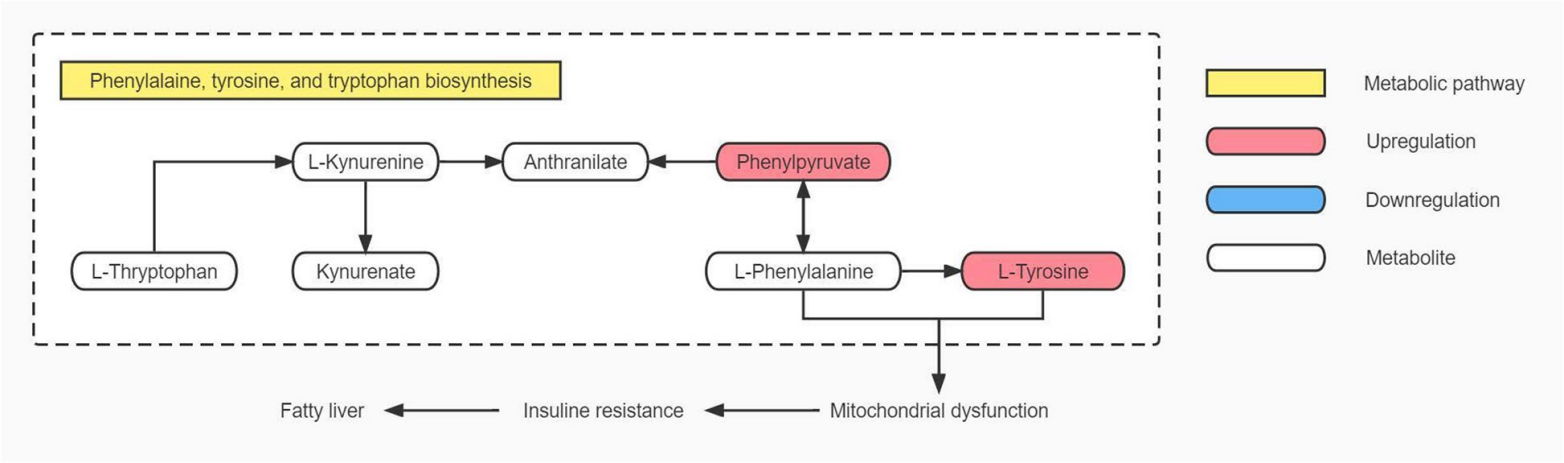
Map of phenylalanine, tyrosine and tryptophan biosynthesis pathway in HUA patients with hyperlipidaemia

Changes in biosynthesis pathway of phenylalanine, tyrosine, and tryptophan lead to mitochondrial dysfunction [33, 34], which plays a major role in insulin resistance [35]. Insulin resistance is closely associated with fatty liver development [36].This claim is well supported by the fact that HUA patients with hyperlipidemia in this study had this pathway altered in 80% of patients with a history of fatty liver disease. Additionally, the differences in serum ALT and AST levels between HUA + HPA group and the NC group were statistically significant but remained within reference values. This suggests that these patients showed no liver impairment, at which point metabolomics showed changes in metabolic pathways associated with fatty liver. This provides insight into whether drugs targeting this pathway can be developed for the early treatment of fatty liver disease. See Fig. 8 for details.

### The relationship between glycine, serine and threonine metabolism pathway changes and CKD in HUA patients with hyperlipidaemia

**Fig. 9:**
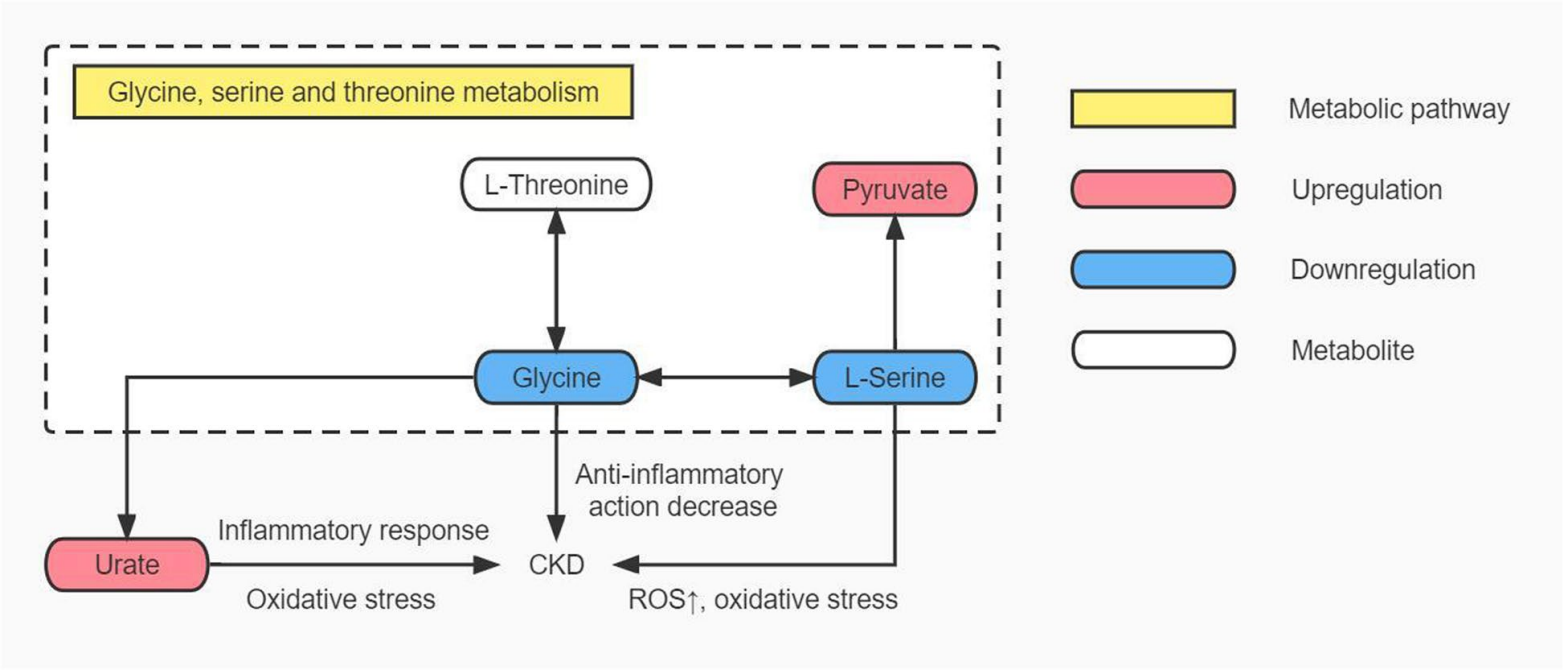
Map of glycine, serine and threonine metabolism pathway in HUA patients with hyperlipidaemia

In this study, serum glycine and serine levels were significantly decreased in HUA patients with hyperlipidaemia, consistent with the metabolomics findings of Luo Y et al. [29] and Mahbub MH et al. [30] in asymptomatic HUA patients. As a result of these changes in the metabolism pathway of glycine, serine and threonine. In addition to its role in uric acid synthesis, this pathway is also involved in inflammatory responses and oxidative stress via glycine and serine [37–39]. Inflammation and oxidative stress are closely correlated with the occurrence of CKD caused by HUA [40]. Changes in glycine, serine and threonine metabolism pathway in HUA patients with hyperlipidaemia promote uric acid synthesis, exacerbating oxidative stress and inflammatory responses, as well as contributing to CKD development. Although there was no abnormal renal function in HUA patients with hyperlipidaemia in this study, the metabolomics results showed changes in this pathway. This should be considered, and in-depth research can be carried out as an entry point in the future. See Fig. 9 for details.

### Comparisons with other studies and what does the current work add to the existing knowledge

Current studies on the relationship between HUA and hyperlipidaemia have mostly been epidemiologically investigated in terms of risk factors [41, 42]. Metabolomics studies have tended to describe metabolic changes in patients with HUA or hyperlipidaemia without exploring their intrinsic associations [24, 43]. This study validates previous findings in human and rat models of HUA and provides a preliminary explanation of the mechanisms of hyperlipidaemia that are caused by HUA in terms of metabolic pathways. Much of the research on HUA associated with hyperlipidaemia and related diseases has also been risk factor analysis [13–15]. This study used metabolomics to analyse changes in serum metabolites in this group of patients and to show the association between changes in metabolic pathways and their associated diseases to provide new insights for future research.

### Strengths and limitations

Metabolomics was used to analyse differences in serum metabolites between HUA patients and HUA patients with hyperlipidaemia and normal control individuals to avoid metabolic differences between animal models and humans. This is the first study of metabolomics in HUA patients with hyperlipidaemia that excludes interference with blood pressure, blood sugar, and a wide range of diseases, providing a true and reliable picture of the metabolic changes associated with this condition.

The HUA patients and HUA with hyperlipidaemia patients in this study were mostly male, which may be connected with the fact that the participants were mostly young adults. The prevalence of HUA in this age group is significantly higher in males than in females [44]. As primary mass spectrometry was used to detect samples in this study, the identification of substances corresponding to some mass spectra is not very accurate, and the results may be biased. The study population included physical examination patients, and their drug history and diet were not included in the study.

## Conclusions

This study found that the incidence rate of hyperlipidaemia in the HUA population was higher than that in the general population. This study used a UHPLC‒MS-based metabolomics method to study the changes in serum metabolic characteristics of HUA patients and HUA patients with hyperlipidaemia and found dozens of abnormal small molecule metabolisms, including carbohydrates, lipids, and amino acids. Pathway analysis showed that the linoleic acid, alanine, aspartate and glutamate metabolism pathways were closely related to HUA-induced hyperlipidaemia. The phenylalanine, tyrosine and tryptophan biosynthesis metabolism pathway exert a significant role in the progression of HUA combined with hyperlipidaemia to fatty liver. HUA patients with hyperlipidaemia had changes in the glycine, serine and threonine metabolism pathway, which may eventually lead to CKD.

When TG and TC levels are normal in HUA patients, linoleic acid metabolites change. These findings provide convincing evidence for the claim that HUA causes hyperlipidaemia and demonstrate the prospect of using metabolomics technology to carry out research on the mechanism of the linoleic acid metabolism pathway. Dominika Maciejewska's study [25] showed that linoleic acid metabolites can help differentiate the early stages of NAFLD. This suggests that not only mechanistic speculation but also linoleic acid metabolism may exert a vital role in early diagnosis of the disease.

This study found that the changes in amino acid metabolism in asymptomatic HUA patients and HUA patients with hyperlipidaemia were closely related to the onset of subsequent diseases. The pathogenesis of the disease was explained from the perspective of small molecules, which provided a direction for subsequent research. Validation of targeted metabolomics and metabolomics multigenomic studies combining genomics, transcriptomics, and proteomics can be performed in the future to systematically and comprehensively explain the impact of metabolic pathways on associated diseases.

## Data Availability

The datasets used and/or analysed during the current study are available from the corresponding author upon reasonable request.

## Abbreviations

AA: Arachidonic acid
ALT: Alanine aminotransferase
AST: Aspartate aminotransferase
BMI: Body mass index
BUN: Blood urea
CKD: Chronic kidney disease
DBP: Diastolic blood pressure
eGFR: Estimated glomerular filtration rate
EP: Eppendorf
FPG: Fasting plasma glucose
HDLc: High-density lipoprotein cholesterol
HMDB: Human metabolome database
HODE: Hydroxyoctadecadienoic acid
HPODE: Hydroperoxyoctadecadienoic acid
HUA: Hyperuricaemia
HUA + HPA: Hyperuricaemia with hyperlipidaemia
KEGG: Kyoto encyclopedia of genes and genomes
LDLc: Low-density lipoprotein cholesterol
NAFLD: Nonalcoholic fatty liver disease
NC: Normal control
OPLS-DA: Orthogonal partial least-squares discrimination analysis
PCA: Principal component analysis
PLS-DA: Partial least-squares discrimination analysis
ROS: Reactive oxygen species
SBP: Systolic blood pressure
SUA: Serum uric acid
UHPLC‒MS: Ultrahigh-performance liquid chromatography-tandem mass spectrometry
TC: Total cholesterol
TG: Triglyceride
VIP: Variable importance in the projection
VLDLc: Very low-density lipoprotein cholesterol
