# Correction: Metabolic pathway analysis of hyperuricaemia patients with hyperlipidaemia based on high-throughput mass spectrometry: a case–control study

**DOI:** 10.1186/s12944-023-01785-4

**Published:** 2023-02-04

**Authors:** Xue Wei, Xiaodong Jia, Rui Liu, Sha Zhang, Shixuan Liu, Jing An, Lei Zhou, Yushi Zhang, Yuanning Mo, Xiao Li

**Affiliations:** 1 Tianjin Union Medical Center, Tianjin Medical University, Tianjin, 300070 China; 2 Tianjin Union Medical Centre, Tianjin, 300121 China; 3 Tianjin Yunjian Medical Technology Co., Ltd., Tianjin, China

**Correction: Lipids Health Dis 21, 151 (2022)**

**https://doi.org/10.1186/s12944-022-01765-0**

Following publication of the original article [1], the author requested to update the acknowledgment section.

The original article has been updated.
